# Cervical Ectropion and Intra-Uterine Contraceptive Device (IUCD): a five-year retrospective study of family planning clients of a tertiary health institution in Lagos Nigeria

**DOI:** 10.1186/1756-0500-7-946

**Published:** 2014-12-23

**Authors:** Kikelomo Ololade Wright, Ahmadu Shehu Mohammed, Olajumoke Salisu-Olatunji, Yetunde Abiola Kuyinu

**Affiliations:** Department of Community Health and Primary Health Care, Lagos State University College of Medicine, Ikeja, Lagos Nigeria; Department of Community Health and Primary Health Care, Lagos State University Teaching Hospital (LASUTH), Ikeja, Lagos Nigeria

**Keywords:** Cervical, Ectropion, Intra-uterine contraceptive device

## Abstract

**Background:**

Cervical ectropion (also known as cervical erosion) is a common finding on routine pelvic examination during the fertile years. The decision to treat or not remains controversial. According to studies in support of routine treatment of cervical erosion, there is a possible relationship between squamous metaplasia and squamous cell carcinoma of the cervix. To determine the prevalence of cervical ectropion and associated risk factors among clients with intra-uterine contraceptive devices (IUCDs) attending a family planning clinic of a tertiary health institution in Lagos, Nigeria.

**Methods:**

A 5-year retrospective study was conducted by assessing existing clinic records from years 2007–2011. Clients with IUCDs undergo routine pelvic examination during check-up visits. A total of 628 clients’ records were seen within the stated time frame. This study was approved by the ethical committee of the Lagos State University Teaching Hospital (LASUTH) and the collected data were analyzed using SPSS version 19.0.

**Results:**

The mean age of the IUCD users was 34.7 ± 6.52 years, while 517 (82.3%) had secondary education. On routine pelvic examination, seventy-nine clients (12.6%) had cervical ectropion. Thirty-nine (6.2%) clients had presented with a history of abnormal vaginal bleeding while 12.1% had vaginal discharge. Treatments offered to cases of cervical ectropion include cervical painting with gentian violet (89.9%) and antibiotics prescription (58.2%). On bivariate analysis, previous hormonal contraceptive use (P = 0.041) and vaginal discharge (P < 0.001) were significantly associated with developing cervical ectropion. Clients with ectropion were significantly more likely to receive prescriptions for antibiotics (P < 0.001).

**Conclusion:**

Less than one fifth of the clients had cervical erosion. However, routine pelvic examination could aid the detection and control of latent reproductive health problems such as cervical ectropion which may require further investigations for example, pap smears, to exclude potentially lethal conditions and to determine appropriate treatment modality.

## Background

Cervical ectropion which is also called cervical ectopy or erosion occurs when eversion of the endocervix exposes columnar epithelium to the vaginal milieu [1]. The prevalence of cervical ectropion ranges from 17% to 50% [1–5]. A prevalence study in China among female users of family planning services reported that 43.2% of the women had cervical ectropion [6]. Another study on women using oral contraceptive pills (OCPs) and IUCDs in Benghazi, Libya also observed that cervical ectropion was the commonest gynecological disorder among the women (54.9%) [7]. Ectropion is considered to be a common physiological condition in adolescents and pregnant women [1, 8, 9].

Cervical ectropion has been associated with both the combined oral contraceptive pill and chlamydia trachomatis [1, 8, 10, 11]. In a study among women attending a sexually transmitted diseases clinic in USA, cervical ectropion was positively associated with oral contraception and Chlamydia trachomatis infection [8]. Inflammation and trauma have also been implicated in the etiology of cervical ectropion [8, 12]. This is important considering the fact that IUCD usage could be linked to inflammation and trauma of the cervix. In view of the fact that vaginal discharge is a common symptom of IUCD users, [9] changing the method of contraception is a common practice for women who are unable to accept the increase in physiological discharge associated with cervical ectropion [13].

Reproductive tract infection is one of the major complications caused by prolonged usage of IUCDs [14]. A Turkish study showed that 14.7% of women using IUCDs had cervical ectropion. Cervical ectropion and vaginal discharge were also found to be significantly higher among IUCD users compared with women on OCPs [15].

Furthermore, ectropion has been found in 5% to 25% of women presenting with postcoital bleeding [16]. Differentiating between cervical ectropion and cervical intraepithelial neoplasia macroscopically is difficult [17]. In addition, available literature concerning treatment for cervical ectropion can be described as controversial [12, 13]. Some arguments advanced in support of routine treatment include the relationship between squamous metaplasia and induction of squamous cell carcinoma of the cervix [18]. Pre-cancerous lesions often develop at the squamous-columnar junction, hence theoretically, treating cervical ectropion may prove to be protective against the incidence of cervical cancer [18]. Proponents of routine treatment have also argued that some sexually transmitted microorganisms such as Chlamydia trachomatis and Neisseria gonorrhea preferentially infect glandular epithelium [10, 15, 19]. Thus ectropion would, by exposing this epithelium, aid an infective process [10, 15, 19]. Several treatment modalities have been used for cases of ectropion including use of antibiotics, microwave tissue coagulation, laser coagulation, infrared light, an interferon-alpha suppository, electrocautery or cryotherapy [2, 12, 13, 20, 21].

Based on the fore-going, the aim of this study was to determine the prevalence of cervical ectropion and associated risk factors among clients with IUCDs of a family planning clinic in a tertiary health institution in Lagos Nigeria.

## Methods

A retrospective study was conducted by assessing existing clinic records between years 2007–2011. Clients with IUCDs undergo routine pelvic examination during check-up visits. A total of 628 clients using IUCDs were seen within the stated time frame. Clinical records of all 628 clients were assessed. Data from the clinical records were collected using a register on the following information: socio-demographic and gynecological characteristics of the clients, gynecological symptoms, presence or absence of ectropion and the type of treatment offered. The collected data were analyzed using SPSS version 19.0 for windows. Descriptive statistics were calculated for variables. Chi square test and multiple logistic regression tests were used to test for association between variables with level of significance set at P < 0.05.

This study was approved by the ethical committee of the Lagos State University Teaching Hospital (LASUTH).

### Study limitations

1. Years 2009–2011 were characterized by periodic industrial actions accounting for the drop in clientele.
2. Furthermore, the obstetrics and gynecology clinics within the hospital complex have been undergoing structural renovation since year 2010 reducing the capacity and services of the family planning clinic to the bearest minimum in an improvised mini-clinic.
3. The study was retrospective; relying on records, with no control over exposure or outcome. It was also hospital-based and may not be readily generalizable to the Nigerian population. However, it provides useful information considering the paucity of research works on cervical ectropion in Nigeria, in addition to highlighting the need for future studies using stronger study designs.

## Results

The majority (88.2%) of the clients were seen between 2007 and 2008. (Table 1). The age of the IUCD users ranged between 16 and 58 years with a mean of 34.7 ± 6.5 years, while 517 (82.3%) had secondary education, the majority (81.4%) were Christians (Table 2).

**Table 1:** **Frequency distribution of clients by year of registration at family planning clinic**

Year	Frequency	Percentage (%)
2007	227	36.1
2008	327	52.1
2009	18	2.9
2010	25	4.0
2011	31	4.9
**Total**	**628**	**100.0**

**Table 2:** **Socio-demographic characteristics of clients**

Characteristic	Frequency	Percentage (%)
**Age group (years)**		
15 - 20	4	0.6
21 - 25	32	5.1
26 - 30	144	22.9
31 - 35	188	29.9
36 - 40	143	22.8
>40	113	18.0
No data	4	0.6
**Total**	**628**	**100.0**
**Educational level**		
None	17	2.7
Primary	35	5.6
Secondary	517	82.3
Tertiary	46	7.3
No data	13	2.1
**Total**	**628**	**100.0**
**Religion**		
Christianity	511	81.4
Islam	103	16.4
No data	14	2.2
**Total**	**614**	**100.0**

Age (Mean = 34.7 years, Median = 34 years, Mode = 35 years, Standard Deviation = 6.52). The majority (75.6%) of respondents were between ages 26 and 40 years conforming averagely to the reproductive age range.

The mean parity of clients using IUCD was 3.2 ± 1.5, with a parity of 3–5 amongst 58.4% of the clients (Table 3). Four hundred and one (65.3%) clients using IUCD had never used any modern contraception previously while 131 (20.9%) had used hormonal contraceptives in the past (Table 3). Most (90.3%) clients had a 25–30 day menstrual cycle while 570 (90.8%) had their menstruation lasting for 3–5 days. Thirty-nine (6.2%) clients had history of abnormal vaginal bleeding while 12.1% had vaginal discharge (Table 4).

**Table 3:** **Gynecological characteristics of clients**

Characteristic	Frequency	Percentage (%)
**Parity**		
Nulliparous	4	0.6
1 - 2	207	33.0
3 - 5	367	58.4
>5	38	6.1
No data	12	1.9
**Total**	**628**	**100.0**
**Previous contraceptive method**		
None	410	65.3
Natural	8	1.3
Barrier	50	8.0
Oral	40	6.4
Injectable	77	12.3
Implant	14	2.2
No data	29	4.6
**Total**	**628**	**100.0**
**History of hormonal contraceptives use**		
Yes	131	20.9
No	468	74.5
No data	29	4.6
**Total**	**628**	**100.0**

Parity (Mean = 3.19, Median = 3, Mode = 3, Standard Deviation = 1.51).

**Table 4:** **Gynecological symptoms presented by clients**

Symptom	Frequency	Percentage (%)
**Abnormal bleeding**		
Yes	39	6.2
No	589	93.8
**Total**	**628**	**100.0**
**Vaginal discharge**		
Yes	76	12.1
No	553	87.9
**Total**	**628**	**100.0**

**Table 5:** **Prevalence of cervical erosion among clients**

Presence of cervical erosion	Frequency	Percentage (%)
Yes	79	12.6
No	549	87.4
**Total**	**628**	**100.0**

Pelvic examination revealed that 79 (12.6%) clients had cervical ectropion (Table 5).

Treatments offered to cases of cervical ectropion include cervical painting with gentian violet paint (89.9%) and antibiotics (58.2%). Forty two clients who had cervical ectropion received both cervical painting with gentian violet and antibiotics (Table 6).

**Table 6:** **Treatment received by clients with cervical erosion (N = 79)**

Treatment	Yes	No	Chi square
	No. (%)	No. (%)	P value
**GV painting**	71 (89.9)	8 (10.1)	X^2^ = 28.3 df = 2
**Antibiotics**	46 (58.2)	33 (41.8)	95% CI = 0.02 to 5.99
**GV paint with antibiotics**	42 (53.2)	37 (46.8)	P < 0.001

**Table 7:** **Association between clients’ age and cervical erosion**

Age group (Years)	Cervical erosion			Chi square P value
	Yes frequency (%)	No frequency (%)	Total frequency (%)	
15 - 20	0 (0.0)	4 (100.0)	4 (100)	
21 - 25	5 (15.6)	27 (84.4)	32 (100)	X^2^ = 5.44
26 - 30	15 (10.4)	129 (89.6)	144 (100)	df = 5
31 - 35	18 (9.6)	170 (90.4)	188 (100)	95% CI = 0.554 to
36 - 40	21 (14.7)	122 (85.3)	143 (100)	11.07
>40	19 (16.8)	94 (83.2)	113 (100)	P = 0.357
**Total**	**78 (12.5)**	**546 (87.5)**	**624 (100)**	

There was no statistically significant association between age group of clients and presence of cervical erosion (P>0.05, Table 7). On bivariate analysis, previous hormonal contraceptive use (P = 0.041) and vaginal discharge (P < 0.001) were significantly associated with developing cervical ectropion (Tables 8 and 9). Clients with ectropion were more likely to receive prescriptions for antibiotics (P < 0.001).

**Table 8:** **Association between gynecological history and cervical erosion**

Gynecological history	Cervical erosion			Chi square P value
	Yes frequency (%)	No frequency (%)	Total frequency (%)	
**Parity**				
Nulliparous	1 (25.0)	3 (75.0)	4 (100)	X^2^ = 7.23
1 - 2	16 (7.7)	191 (92.3)	207 (100)	df = 3
3 - 5	55 (15.0)	312 (85.0)	367 (100)	95% CI = 0.115 to
>5	6 (15.8)	32 (84.2)	38 (100)	7.81
**Total**	**78 (12.7)**	**538 (87.3)**	**616 (100)**	P = 0. 065
**Previous hormonal contraceptive**				
No	51 (10.9)	417 (89.1)	468 (100)	X^2^ = 4.19
Yes	23 (17.6)	108 (82.4)	131 (100)	df = 1
**Total**	**74 (12.4)**	**525 (87.6)**	**599 (100)**	95% CI = 0.0 to 3.84 P = 0.041

**Table 9:** **Association between gynecological symptoms and cervical erosion**

Gynecological symptom	Cervical erosion			Chi square P value
	Yes frequency (%)	No frequency (%)	Total frequency (%)	
**Abnormal bleeding**				
No	75 (12.7)	514 (87.3)	589 (100)	X^2^ = 0.204
Yes	4 (10.3)	35 (89.7)	39 (100)	df = 1
**Total**	**79 (12.6)**	**549 (87.4)**	**628 (100)**	95% CI = 0.0 to 3.84 P = 0. 806
**Vaginal discharge**				
No	55 (10.0)	497 (90.0)	552 (100)	X^2^ = 28 .38
Yes	24 (31.6)	52 (68.4)	76 (100)	df = 1
**Total**	**79 (12.6)**	**549 (87.4)**	**628 (100)**	95% CI = 0.0 to 3.84 P <0.001

Multiple logistic regression analysis was employed to predict the probability that a client would have cervical ectropion. The predictor variables were client’s age, parity, history of previous use of hormonal contraceptives, presence of abnormal vaginal bleeding and presence of vaginal discharge. A test of the full model versus a model with intercept only was statistically significant, χ2 = 33.337, d.f. = 5, p < 0.001.

Table 10 shows the Multiple logistic regression coefficient, Wald test, odds ratio and 95% confidence interval of odds ratio for each of the predictors. Employing a P < 0.05 criterion of statistical significance, only presence of vaginal discharge had a statistically significant partial effect on the presence of cervical ectropion. The odds ratio for presence of vaginal discharge indicates that when holding all other variables constant, a client with vaginal discharge is 2.56 times more likely to have cervical ectropion than is a client without vaginal discharge. Although previous use of hormonal contraceptive was statistically significantly associated with cervical ectropion on bivariate analysis, this significant association was lost after multivariate analysis.

**Table 10:** **Multiple logistic regression predicting cervical ectropion from age, parity, hormonal contraceptives use, abnormal vaginal bleeding, and vaginal discharge**

Predictor	***B***	Wald χ2	***p***	Odds ratio	95% CI for odds ratio	
Age	0.025	1.185	0.276	1.026	0.98	1.074
Parity	0.112	1.214	0.271	1.119	0.916	1.366
Hormonal contraceptives use	0.525	3.229	0.072	1.69	0.953	2.997
Abnormal vaginal bleeding	-0.583	0.851	0.356	0.558	0.162	1.926
Vaginal discharge	1.52	26.433	< 0.001	4.571	2.561	8.158

Omnibus Test of Model Coefficient (*χ2* = 33.337, d.f. = 5, *p* < 0.001).

## Discussion

The prevalence of cervical ectropion among women using IUCDs varies widely in the literature. In China the prevalence in this group of women was 43.2% compared to the 12.6% observed in this study [6]. The prevalence was higher (26%) among IUCD users in a family planning clinic at Edinburgh [1]. In a study to assess the effects of IUCD and oral contraceptives on vaginal flora and epithelium conducted in Turkey however, the prevalence of ectropion among IUCD users was 14.6%, which is close to what has been observed in this study [15]. Notwithstanding the fact that previous studies observed higher prevalence of ectropion among IUCD users, the 12.6% prevalence rate observed in this study indicates that ectropion is a fairly common occurrence in this group of women. A possible explanation for the occurrence of cervical ectopy in IUCD users is the constant friction between the hanging string and the cervical os.

This study observed that women were significantly more likely to have developed an abnormal vaginal discharge if they had cervical ectropion compared with those without ectropion. A previous research has demonstrated an association between use of the IUCD and cervical inflammation and ectropion, either of which could potentially lead to abnormal discharge [4]. Vaginal samples for Trichomonas vaginalis and other sexually transmitted diseases were not obtained as part of this study. Therefore, it was not possible to differentiate vaginal discharge due to pelvic infection from that which may have been solely due to the ectropion. However, it is known that prolonged use of IUCD is associated with increased risk of pelvic infection [15].

Studies have suggested that cervical ectropion is common among those taking estrogen-containing contraceptives [1, 11, 22]. The observation from this study that women who had a previous history of hormonal contraceptive use were more likely to develop ectropion (P < 0.05) however disappeared when other factors were taken into consideration (P = 0.072). This finding is supported by a study which observed that cervical ectropion was more common among IUCD users than hormonal contraceptive users [15]. This suggests therefore that previous use of hormonal contraceptives does not confer additional risk of ectropion to IUCD users.

The tendency towards treating cervical ectropion is strong as has been demonstrated in this study. Most of the clients with ectropion in this study received some form of treatment. Several treatment modalities are currently used in the clinical management of cervical ectropion [13]. However the two modalities used in this study comprise of the use of antibiotics and painting of the cervix with gentian violet. The 12.6% prevalence of ectropion means that a substantial number of women were exposed to antibiotics. A contrary argument on the current treatment modality is that inappropriate use of antibiotics increases the risk of antimicrobial resistance and places an unnecessary financial burden on patients [23, 24]. Moreover, in the context of cervical erosion being a non-infective and physiological condition, it may resolve spontaneously without medical intervention.

## Conclusions

The study findings indicate that cervical ectropion may be a common occurrence among women using IUCD. Reproductive health clinics offer screening opportunities for various groups of women in the society. Therefore, routine pelvic examination should be emphasized for all family planning clients as it could aid the detection and control of latent reproductive health problems such as cervical ectopy which may require further investigations such as microscopy, culture and sensitivity as well as pap smears to exclude potentially lethal conditions and to determine appropriate treatment modality.
